# Antibiotic-Loaded PLA Composites for Local Prevention of Implant-Associated Infections: Comparative Evaluation Against Reference Strains and Clinical Isolates

**DOI:** 10.3390/antibiotics15040373

**Published:** 2026-04-06

**Authors:** Anastassiya Khrustaleva, Azamat Yedrissov, Dmitriy Khrustalev, Irina Losseva, Alyona Lavrinenko, Artyom Savelyev, Vladimir Kazantsev, Marlen Kiikbayev, Polina Rusyaeva, Kristina Perepelitsyna, Aigerim Donenbaeva

**Affiliations:** 1 School of Pharmacy, Karaganda Medical University, Gogol Street 40, Karaganda 100008, Kazakhstan; anasteishin_2009@mail.ru (A.K.); savelevrtm@gmail.com (A.S.); rusyaevapolina@mail.ru (P.R.);; 2 Scientific Research Laboratory, Karaganda Medical University, Gogol Street 40, Karaganda 100008, Kazakhstan

**Keywords:** polylactic acid (PLA), antibacterial composites, 3D printing, implant-associated infections, local antibiotic delivery, clinical isolates, MRSA, *Pseudomonas aeruginosa*, antibiotic resistance

## Abstract

**Background/Objectives**: Implant-associated infections remain among the most severe and clinically challenging complications in contemporary orthopedics, largely due to the formation of persistent bacterial biofilms and the limited penetration of systemically administered antibiotics into the tissue–implant interface. In this context, local antibacterial functionalization of implantable materials represents a promising strategy for the prevention of early infectious complications. The objective of this study was to develop and comparatively evaluate the antimicrobial performance of PLA-based composites loaded with antibiotics from different pharmacological classes, with a view toward their potential application in individualized 3D-printed implants. **Methods**: Polylactic acid (PLA)-based composites incorporating gentamicin, ciprofloxacin, doxycycline, and vancomycin were fabricated using thermal processing under conditions compatible with extrusion and fused filament fabrication. Physicochemical characterization (FTIR, TGA, SEM) was performed to assess the structure and morphology of the composites, and in vitro antibiotic release studies were conducted. Antimicrobial activity was evaluated using an agar diffusion assay against ATCC reference strains and clinical isolates of methicillin-susceptible and methicillin-resistant *Staphylococcus aureus* (MSSA and MRSA), *Klebsiella pneumoniae*, and *Pseudomonas aeruginosa* (*n* = 10 per species). The antibacterial performance of the composites was evaluated in comparison with standard commercial antibiotic disks used as qualitative reference controls. **Results**: Antibiotic-loaded PLA composites exhibited consistent and reproducible antibacterial activity, markedly exceeding that of neat PLA. The broadest activity spectrum was observed for PLA–ciprofloxacin (≈29–36 mm) and PLA–gentamicin (≈25–27 mm), which effectively inhibited both Gram-positive and Gram-negative clinical isolates, including MRSA and *P. aeruginosa*. PLA–vancomycin retained selective activity against staphylococci (≈14–15 mm), whereas PLA–doxycycline demonstrated limited efficacy against Gram-negative pathogens. Physicochemical analysis confirmed successful incorporation of antibiotics without detectable degradation of the polymer structure, while release studies demonstrated sustained antibiotic release from the composite materials. Importantly, the expected pharmacological activity profiles of the antibiotics were preserved after incorporation into the polymer matrix and subsequent high-temperature processing. **Conclusions**: The results demonstrate the feasibility of integrating clinically relevant antibiotics into a thermoplastic PLA matrix while preserving their selective antimicrobial activity following processing compatible with extrusion and additive manufacturing. The proposed PLA-based composites can be regarded as elements of a pharmacologically tunable antibacterial platform, offering a rationale for the development of context-dependent, biodegradable, 3D-printed implants for the local prevention of implant-associated infections in the setting of increasing antimicrobial resistance.

## 1. Introduction

Infections associated with bone implants, scaffolds, and fixation devices remain one of the most serious challenges in contemporary clinical practice, as they lead to repeated surgical interventions, prolonged hospitalization, increased healthcare costs, and a higher risk of patient disability [1,2,3]. Sources of implant contamination include both the patient’s endogenous microbiota and the hospital environment, encompassing operating rooms, medical personnel, instruments, and invasive procedures [4]. The risk of infection is particularly high in orthopedic and trauma surgery, where the implant is in direct contact with tissues and blood, creating favorable conditions for microbial adhesion and biofilm formation [5].

The main causative agents of implant-associated infections are Gram-positive cocci, primarily *Staphylococcus aureus* and coagulase-negative staphylococci, which account for approximately 40–60% of periprosthetic infections, with a significant contribution from Gram-negative bacteria such as *Klebsiella* spp. (up to 26%) and *Pseudomonas aeruginosa* (up to 16%) [6]. The incidence of infections associated with orthopedic implants is estimated at approximately 6–13% of surgical interventions [7]. A major concern is the high prevalence of antibiotic-resistant strains, including methicillin-resistant *S. aureus* (MRSA), which may exceed 40–60% of clinical isolates, and extended-spectrum β-lactamase (ESBL)-producing Enterobacterales, reaching ~60–70% [8]. These pathogens readily adhere to medical surfaces and form biofilms, substantially increasing their tolerance to antimicrobial agents and host immune defenses [7]. Together, these factors highlight the susceptibility of implantable orthopedic devices to colonization by nosocomial flora and support the development of materials providing local antimicrobial protection. The global impact of antimicrobial resistance further emphasizes the importance of this problem. According to the Global Burden of Disease study, bacterial AMR was associated with approximately 4.7 million deaths worldwide in 2021, including more than 1.1 million deaths directly attributable to AMR [9,10].

In this context, local antibiotic delivery is considered a promising strategy for the prevention and treatment of implant-associated infections and involves the targeted administration of antimicrobial agents directly to the implantation site using various carriers, including antibiotic-loaded bone cements, calcium-based matrices, and local perfusion systems [11,12]. This approach enables the establishment of high local antibiotic concentrations with minimal systemic exposure, which is critically important for the eradication of bacteria within biofilms, whose tolerance markedly exceeds that of planktonic forms.

Despite the clinical efficacy of existing approaches to local antibiotic delivery, such systems have several limitations, including a lack of biodegradability, the need for secondary surgical interventions to remove temporary carriers, and limited opportunities for fine-tuning drug release profiles and integration with bone regeneration processes. In this regard, biodegradable polymeric materials are of particular interest as platforms for local antibiotic therapy in orthopedics.

Among these, polylactic acid (PLA) is widely used owing to its favorable combination of biocompatibility, predictable degradation kinetics, and technological versatility, including compatibility with extrusion and additive manufacturing processes [13]. Incorporation of antibiotics into the PLA matrix enables the development of multifunctional composite materials that combine mechanical support with controlled release of therapeutic agents directly at the implantation site [11].

In recent years, the development of 3D-printable filaments based on biodegradable polymers has been regarded as one of the key enablers of personalized medicine in orthopedics and traumatology, allowing the fabrication of implantable constructs with individualized geometry and tailored porous architectures [14]. However, according to recent reviews, the vast majority of studies in this field have focused on enhancing the osteoconductive and osteogenic properties of materials through the incorporation of hydroxyapatite, β-tricalcium phosphate, and bioglasses, whereas the development of 3D-printed polymer composites with deliberately engineered antimicrobial functionality remains substantially underexplored and accounts for no more than 10% of the total body of publications [15]. This imbalance contrasts with the clinical relevance of implant-associated infections and highlights the need to integrate antibacterial functionality into the design of 3D-printed implantable materials.

The performance of such systems is, however, largely determined by the physicochemical and pharmacological properties of the incorporated antibiotics, including molecular weight, solubility, thermal stability, and antimicrobial spectrum. Antibiotics from different pharmacological classes—aminoglycosides (gentamicin), glycopeptides (vancomycin), fluoroquinolones (ciprofloxacin), and tetracyclines (doxycycline)—differ substantially in their mechanisms of action and activity profiles against Gram-positive and Gram-negative microorganisms [11,16]. These differences may critically affect their compatibility with the polymer matrix, spatial distribution within the composite, release kinetics, and, ultimately, the overall antimicrobial performance of local delivery systems.

Despite the active development of polymer-based platforms for local antibiotic therapy, systematic comparative studies evaluating antibiotics from different pharmacological classes within the same polymer matrix against clinically relevant pathogens are currently lacking. Most published studies focus either on a single antibiotic or on testing against reference laboratory strains, which substantially limits the clinical interpretability and practical relevance of the reported findings [11,16]. In contrast, direct comparison of agents with different mechanisms of action—glycopeptides, aminoglycosides, tetracyclines, and fluoroquinolones—within a single PLA platform provides a rational basis for identifying optimal strategies for local antibacterial protection of implants.

In the present study, a comprehensive comparative evaluation of PLA composites modified with gentamicin, vancomycin, doxycycline, and ciprofloxacin was performed for the first time. The antibacterial activity of the materials was assessed against both standard ATCC reference strains and clinical isolates (*n* = 10 for each species), including MRSA and resistant Gram-negative bacteria associated with healthcare-associated infections. This approach enables a shift from formal laboratory testing toward a clinically oriented assessment of the potential of polymer-based local antibiotic delivery systems.

The objective of this study was to comparatively evaluate the antimicrobial efficacy of PLA composites containing antibiotics from different pharmacological classes against reference and clinical strains of microorganisms associated with implant-associated and healthcare-associated infections, in order to substantiate rational design principles for biodegradable antibacterial implantable materials for orthopedic applications.

## 2. Results

### 2.1. Physicochemical Characterization of PLA Composites

#### 2.1.1. FTIR Characterization

**Figure 1:**
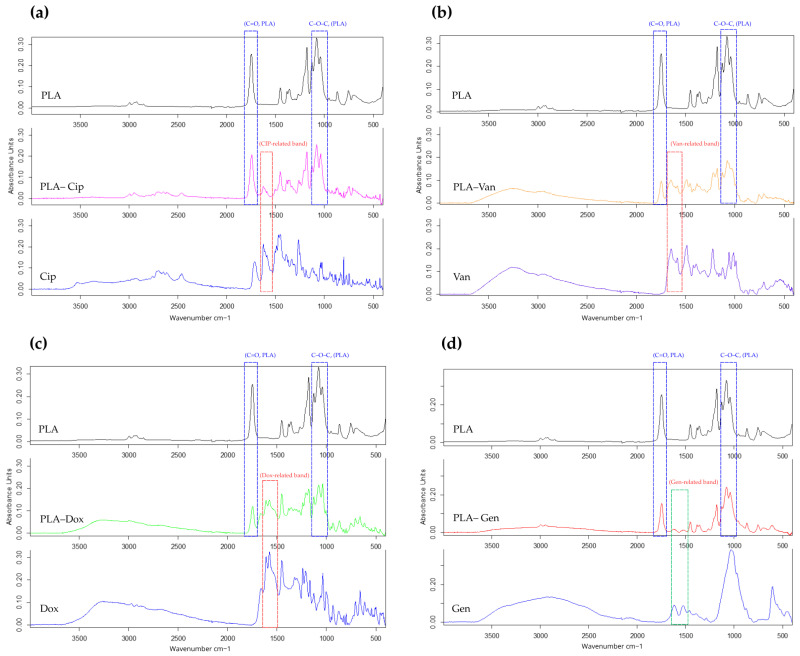
FTIR spectra of neat PLA, pure antibiotics, and PLA-based composites containing (**a**) ciprofloxacin, (**b**) vancomycin, (**c**) doxycycline, and (**d**) gentamicin. The characteristic PLA bands (C=O and C–O–C) are highlighted in blue, while antibiotic-related spectral contributions are indicated by dashed boxes (red/green). The appearance of additional bands in the composites confirms the incorporation of antibiotics into the PLA matrix.

The chemical structure of PLA-based composites containing ciprofloxacin, doxycycline, vancomycin, and gentamicin was investigated using Fourier-transform infrared (FTIR) spectroscopy (Figure 1). For improved comparison, the FTIR spectra of neat PLA, pure antibiotics, and the corresponding PLA–antibiotic composites are presented together in a single figure.

The FTIR spectra of all PLA–antibiotic composites were predominantly characterized by the absorption bands of the PLA matrix. In particular, a strong and well-defined peak at approximately 1750 cm^−1^, corresponding to the ester carbonyl (C=O) stretching vibration, was observed in all samples, indicating preservation of the polymer backbone after incorporation of the antibiotics. Additionally, characteristic PLA bands in the regions of 2995–2945 cm^−1^ (C–H stretching), 1450–1380 cm^−1^ (CH_3_ bending), and 1180–1080 cm^−1^ (C–O–C stretching) were retained in all composite spectra.

Despite the dominant contribution of PLA, additional spectral features associated with the incorporated antibiotics were observed, although their visibility depended on the chemical structure of the respective compound.

For the PLA–ciprofloxacin composite, characteristic spectral contributions were observed in the region of 1700–1600 cm^−1^, corresponding to quinolone-related vibrations, as well as in the fingerprint region, confirming the presence of ciprofloxacin within the polymer matrix. In the PLA–doxycycline and PLA–vancomycin composites, a noticeable broadening of the absorption band in the 3600–3200 cm^−1^ region was observed, which can be attributed to overlapping O–H and N–H stretching vibrations of the incorporated antibiotics. In addition, partially retained bands associated with carbonyl and amide groups were detected, although with reduced intensity due to overlap with PLA signals.

In contrast, the FTIR spectrum of the PLA–gentamicin composite was nearly identical to that of neat PLA. Only a weak and broad absorption band in the region of 3600–3200 cm^−1^ was observed, while characteristic gentamicin peaks were not clearly distinguishable due to significant overlap with the PLA bands and their relatively low intensity.

No new absorption bands or significant shifts in peak positions were detected in any of the composite spectra, indicating that no new covalent bonds were formed during the incorporation process. The results suggest that the antibiotics are incorporated into the PLA matrix predominantly through physical interactions, with possible contributions from intermolecular interactions such as hydrogen bonding.

Overall, the FTIR analysis confirms the successful incorporation of the antibiotics into the PLA matrix while preserving the chemical structure of the polymer and supports the absence of significant chemical transformations during composite formation.

#### 2.1.2. Thermogravimetric Analysis (TGA)

**Figure 2:**
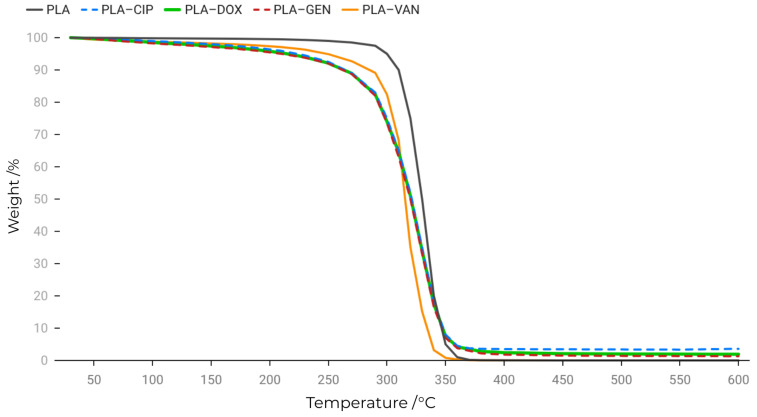
Thermogravimetric (TG) curves of neat PLA and PLA-based composites containing ciprofloxacin (PLA–CIP), doxycycline (PLA–DOX), gentamicin (PLA–GEN), and vancomycin (PLA–VAN), recorded under an inert argon atmosphere.

The thermal behavior of neat PLA and PLA-based composites containing ciprofloxacin, doxycycline, vancomycin, and gentamicin was investigated by thermogravimetric analysis under an inert argon atmosphere (Figure 2; Figure S1).

Neat PLA exhibited high thermal stability, with no significant mass loss observed below approximately 300 °C. The main degradation occurred within a relatively narrow temperature interval of 300–370 °C, indicating a predominantly single-step thermal decomposition process, with negligible residual mass at 600 °C (~0.1%).

As shown in Figure 2, all PLA–antibiotic composites exhibited similar thermal degradation profiles predominantly governed by the PLA matrix. A minor initial mass loss was observed below approximately 280–300 °C, ranging from ~3.5% to ~4.2%, which is likely associated with the removal of adsorbed moisture and low-molecular-weight volatile species originating from the incorporated antibiotics.

The thermogravimetric profiles of the pure antibiotics (Figure S1, Supplementary Materials) indicate that their main thermal degradation occurs at temperatures above approximately 220–260 °C, depending on the compound. These values are significantly higher than the processing temperature used for hot pressing (160–180 °C), indicating that thermal degradation of the antibiotics during composite fabrication is unlikely.

The main degradation step for all composites occurred within a relatively narrow temperature range of approximately 320–380 °C, corresponding to the characteristic thermal decomposition of PLA. The preservation of this predominantly single-step degradation profile indicates that the incorporation of antibiotics does not significantly alter the principal thermal degradation mechanism of the polymer matrix. At the same time, slight differences in thermal stability were observed between the composites, with PLA–vancomycin exhibiting the earliest onset of degradation, as confirmed by the lowest Tonset value (Table S1, Supplementary Materials). A slight broadening of the degradation curves was observed, which can be attributed to overlapping decomposition processes of PLA and the incorporated antibiotics.

The main thermal parameters derived from the TGA curves, including Tonset, T5%, Tmax, and residual mass, are summarized in Table S1 (Supplementary Materials) for quantitative comparison.

Differences between the composites were primarily reflected in the residual mass at 600 °C. The PLA–ciprofloxacin composite exhibited the highest residual mass (~3.6%), followed by PLA–doxycycline (~1.9%) and PLA–gentamicin (~1.3%), whereas the PLA–vancomycin composite showed negligible residue (approximately 0%). Although the pure antibiotics demonstrated substantial residue formation under inert conditions, their contribution in the composites remained limited, which is consistent with their relatively low loading and dispersion within the PLA matrix.

The presence of a small but reproducible residual mass, which is absent in neat PLA, provides indirect but consistent evidence of successful incorporation of the antibiotics into the polymer matrix.

Overall, the TGA results confirm the thermal compatibility of the selected processing conditions with the incorporated antibiotics and support their successful incorporation into the PLA matrix.

#### 2.1.3. Scanning Electron Microscopy (SEM) Analysis

**Figure 3:**
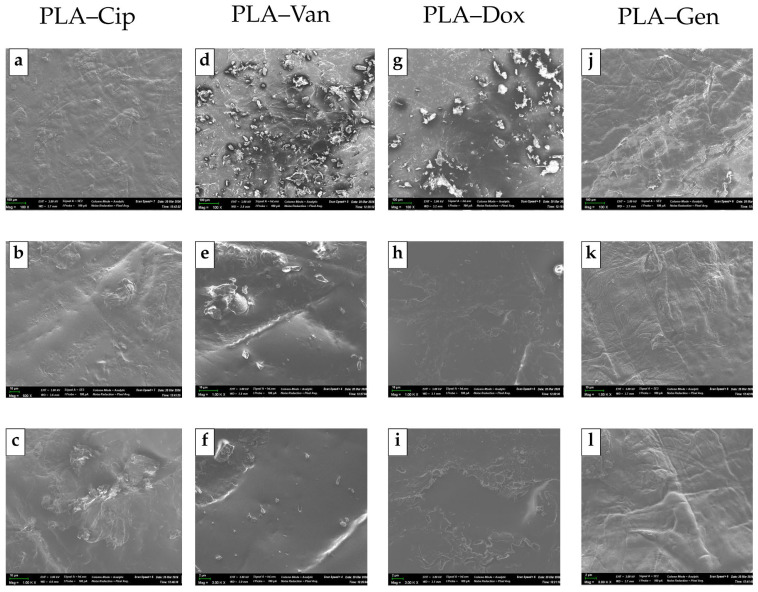
SEM micrographs of PLA-based composites containing different antibiotics: (**a**–**c**) PLA–ciprofloxacin (PLA–Cip), (**d**–**f**) PLA–vancomycin (PLA–Van), (**g**–**i**) PLA–doxycycline (PLA–Dox), and (**j**–**l**) PLA–gentamicin (PLA–Gen) at different magnifications.

The surface morphology of PLA-based composites containing ciprofloxacin, vancomycin, doxycycline, and gentamicin was examined by scanning electron microscopy (Figure 3).

The presented SEM micrographs are representative of the overall morphology observed for each formulation.

The PLA–ciprofloxacin composite exhibited a continuous polymer matrix with dispersed microstructural features (Figure 3a–c). At higher magnifications, small irregular domains were observed within the matrix, which may correspond to the dispersed ciprofloxacin phase. These domains were distributed throughout the material, with occasional localized clustering.

The PLA–vancomycin composite demonstrated a more developed surface microrelief (Figure 3d–f). Particulate features of varying size and morphology were observed across the surface, forming locally concentrated regions and contributing to increased surface roughness.

In contrast, the PLA–doxycycline composite displayed a relatively uniform morphology (Figure 3g–i). The surface appeared continuous, with fine and evenly distributed microstructural features, suggesting a more homogeneous dispersion of doxycycline within the PLA matrix.

The PLA–gentamicin composite was characterized by a predominantly homogeneous structure (Figure 3j–l). The surface exhibited a smooth microrelief with features typical of processed polymer materials, including flow-like patterns. No distinct particulate domains were observed at the examined magnifications, which may indicate either a finer dispersion of gentamicin within the matrix or particle sizes below the resolution of the applied magnification.

Overall, all composites retained the structural continuity of the PLA matrix, while the incorporated antibiotics may be present in different dispersed forms depending on their physicochemical properties, such as solubility and interaction with the polymer matrix. It should be noted that some surface irregularities and flow-like features may also be associated with the processing conditions (e.g., hot pressing) and the viscoelastic behavior of the PLA matrix.

These results indicate that the type of incorporated antibiotic influences the surface morphology and apparent dispersion behavior within the PLA matrix, which may be relevant for subsequent material performance.

### 2.2. In Vitro Antibiotic Release

**Figure 4:**
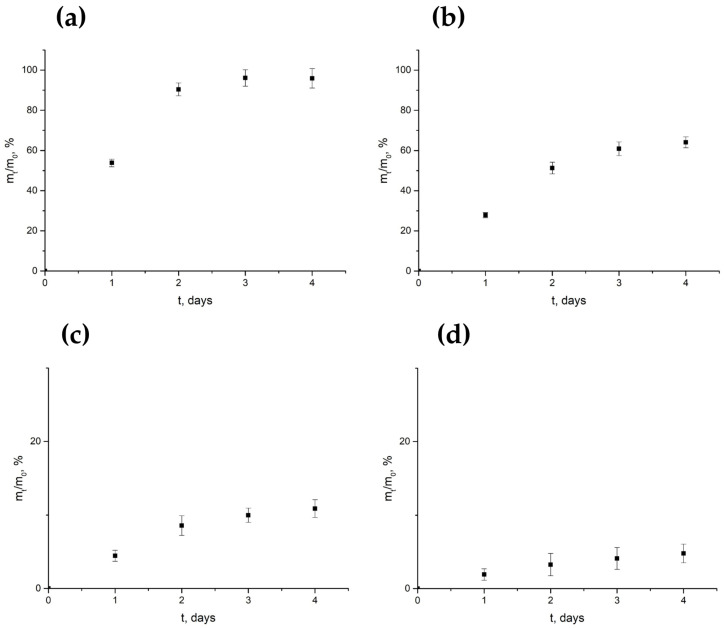
Cumulative release profiles (m_t/m_0) of antibiotics from PLA-based composites over 4 days: (**a**) gentamicin, (**b**) ciprofloxacin, (**c**) vancomycin, (**d**) doxycycline. Data are presented as mean ± SD (*n* = 3).

The cumulative release profiles of the investigated antibiotics from PLA-based composites are presented in Figure 4. The data are shown as mean values with standard deviation (±SD), taking into account complete medium replacement at each sampling point.

Gentamicin exhibited the fastest and most extensive release among all studied antibiotics. A biphasic release profile was observed, with an initial burst phase during the first two days followed by a plateau. Approximately 53.8 ± 1.9% of the loaded antibiotic was released within the first 24 h, increasing to 90.4 ± 3.2% after 48 h. The release reached 95.9–96.1 ± 4.8% by day 3, indicating near-complete elution.

Ciprofloxacin demonstrated a moderately sustained release without reaching a plateau within the 4-day observation period. The cumulative release reached 27.8 ± 1.3% after 24 h, 51.3 ± 2.9% after 48 h, 60.9 ± 3.4% by day 3, and 64.1 ± 2.7% by day 4. The absence of a plateau suggests a prolonged release behavior beyond the studied time frame.

Vancomycin exhibited limited release, reaching only 10.9 ± 1.2% of the loaded amount by day 4. The release profile was characterized by low cumulative values throughout the entire observation period.

Doxycycline showed the lowest release among all investigated antibiotics, with only 4.8 ± 1.3% of the loaded amount released over 4 days. The release profile was characterized by a very slow and nearly linear increase without a pronounced burst phase.

For comparison, antibiotic release from standard commercial discs (without polymer matrix) was also evaluated under the same conditions. In all cases, nearly complete release (98–100%) was observed within the first 24 h, indicating immediate drug availability in the absence of a polymer carrier.

Overall, the obtained release profiles demonstrate substantial differences in release kinetics depending on the type of antibiotic, ranging from rapid and near-complete release (gentamicin) to extremely limited release (doxycycline).

### 2.3. Antimicrobial Activity of PLA Composites Against ATCC Reference Strains

The antimicrobial activity of PLA-based composites against the reference strains *Staphylococcus aureus* ATCC 29313, *Pseudomonas aeruginosa* ATCC 27853, and *Escherichia coli* ATCC 25922 is summarized in Table 1. Neat PLA exhibited no clinically relevant antimicrobial activity, producing only minimal inhibition zones comparable to the disk diameter (6–7 mm), thereby confirming the absence of intrinsic antibacterial properties of the polymer matrix.

For the antibiotic-modified composites, a clear hierarchy of antimicrobial activity was observed, depending on both the nature of the incorporated antibiotic and the microbial species. PLA–ciprofloxacin exhibited the most pronounced effect, producing the largest inhibition zones against all tested strains, with particularly high activity against Gram-negative bacteria (e.g., up to ≈35 mm for *P. aeruginosa* and ≈43 mm for *E. coli*). PLA–gentamicin demonstrated strong and relatively uniform activity against both Gram-positive and Gram-negative strains (25–29 mm).

In contrast, PLA–doxycycline showed selective activity, with pronounced effects against *S. aureus* and *E. coli* (≈26 mm), but was virtually inactive against *P. aeruginosa*, which is consistent with the limited efficacy of tetracyclines against non-fermenting Gram-negative bacteria. PLA–vancomycin exhibited antimicrobial activity exclusively against *S. aureus* (≈16 mm), reflecting the selective spectrum of glycopeptide antibiotics and the lack of activity against Gram-negative bacteria.

**Figure 5:**
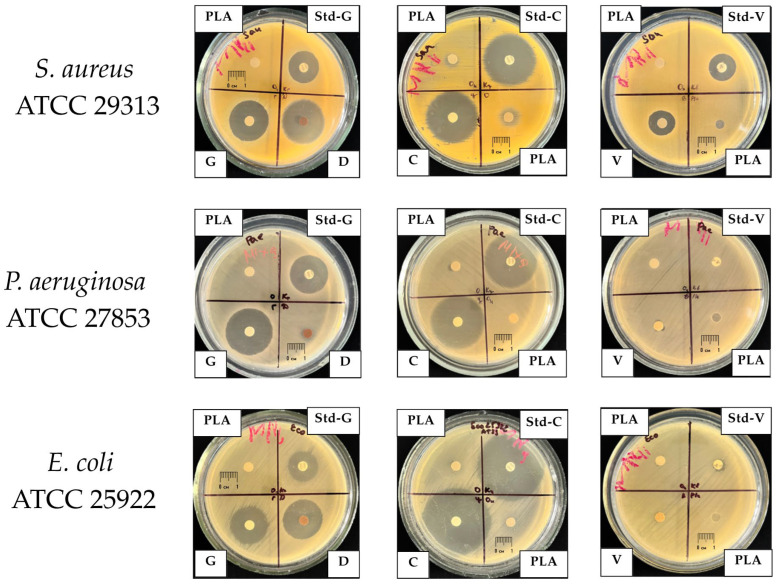
Representative inhibition zones around PLA-based composite disks and commercial antibiotic disks against *S. aureus* ATCC 29313, *P. aeruginosa* ATCC 27853, and *E. coli* ATCC 25922 after 24 h of incubation at 37 °C. Disks are labeled as follows: PLA—neat PLA (negative control); G—PLA–gentamicin; C—PLA–ciprofloxacin; D—PLA–doxycycline; V—PLA–vancomycin; Std-G, Std-C, and Std-V—commercial gentamicin, ciprofloxacin, and vancomycin disks (positive controls), respectively. Clear halos around the disks indicate zones of bacterial growth inhibition.

Representative examples of inhibition zones formed by PLA composites and control samples are shown in Figure 5.

Comparison of the antimicrobial activity of PLA composites with standard antibiotic susceptibility testing disks (BIOANALYSE Limited, Ankara, Turkey) demonstrated comparable levels of antibacterial effects for the corresponding “antibiotic–pathogen” combinations, indicating preservation of the biological activity of antibiotics after incorporation into the polymer matrix and thermal processing.

### 2.4. Antimicrobial Activity Against Clinical Isolates

The antimicrobial activity of PLA-based composites was evaluated against clinical isolates of methicillin-susceptible *Staphylococcus aureus* (MSSA, *n* = 10), methicillin-resistant *Staphylococcus aureus* (MRSA, *n* = 10), *Klebsiella pneumoniae* (*n* = 10), and *Pseudomonas aeruginosa* (*n* = 10) (Table 2).

Comparison of the activity of PLA composites against ATCC reference strains and clinical isolates of the same species (*S. aureus* ATCC 29313 vs. MSSA/MRSA; *P. aeruginosa* ATCC 27853 vs. clinical *P. aeruginosa* isolates) showed that clinical populations were characterized by a broader interstrain variability in inhibition zone diameters, while the overall pharmacological activity profiles of the respective antibiotics were preserved. Mean inhibition zone diameters for clinical isolates were within a comparable range to those observed for ATCC strains; however, dispersion was substantially higher, reflecting the heterogeneity of clinical microbial populations.

Despite the increased variability in responses among clinical isolates, reproducible trends in antimicrobial activity were preserved for individual PLA composites and were consistent with those observed for ATCC reference strains. Thus, PLA–ciprofloxacin yielded high inhibition zone diameters for MSSA and MRSA (approximately 30 mm), as well as for Gram-negative clinical pathogens, reaching maximal values for *P. aeruginosa* (up to ~36 mm), which is consistent with its enhanced activity against Gram-negative bacteria observed in ATCC controls. PLA–gentamicin exhibited comparable activity against all tested clinical isolates (on average ~24–27 mm). PLA–doxycycline demonstrated pronounced activity against clinical MSSA and MRSA isolates (~26–27 mm) and markedly lower activity against Gram-negative pathogens, particularly *P. aeruginosa* (~10 mm). PLA–vancomycin showed antimicrobial activity predominantly against clinical *S. aureus* isolates (~15 mm) and minimal effects against Gram-negative bacteria.

Statistical analysis revealed significant differences in antimicrobial activity among PLA composites for all groups of clinical isolates (Kruskal–Wallis test, *p* < 0.001 for MSSA, MRSA, *K. pneumoniae*, and *P. aeruginosa*). Dunn’s post hoc test with Holm correction showed that, for staphylococcal clinical isolates (MSSA and MRSA), PLA–vancomycin produced significantly smaller inhibition zones than PLA–ciprofloxacin and PLA–gentamicin (*p_adj* < 0.01), and was also inferior to PLA–doxycycline *(p_adj* < 0.01), reflecting differences in the pharmacological profiles of the antibiotics while preserving their selective activity within the polymer matrix. For Gram-negative clinical pathogens (*K. pneumoniae* and *P. aeruginosa*), PLA–ciprofloxacin exhibited significantly larger inhibition zones than PLA–doxycycline (*p_adj* < 0.01), which is consistent with the limited efficacy of tetracyclines against non-fermenting Gram-negative bacteria. The activity of PLA–vancomycin against Gram-negative pathogens was not considered clinically relevant and is consistent with the known lack of activity of glycopeptides against this group of microorganisms. Differences between PLA–ciprofloxacin and PLA–gentamicin were statistically significant for Gram-negative pathogens, whereas for staphylococcal isolates they approached borderline significance after correction for multiple comparisons, indicating comparable activity profiles of these composites within the applied experimental model.

**Figure 6:**
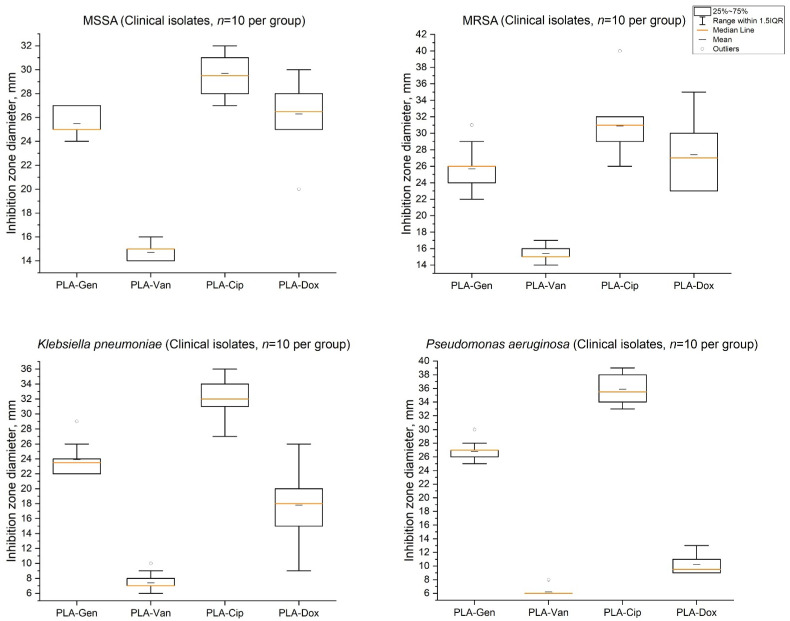
Distribution of inhibition zone diameters for PLA-based composites against clinical isolates of methicillin-susceptible *Staphylococcus aureus* (MSSA), methicillin-resistant *Staphylococcus aureus* (MRSA), *Klebsiella pneumoniae*, and *Pseudomonas aeruginosa* (*n* = 10 per group). Boxplots show the median (central line), interquartile range (25–75%), whiskers indicating the range within 1.5× IQR, and individual outliers. PLA-based composites are denoted as PLA–Gen (gentamicin), PLA–Van (vancomycin), PLA–Cip (ciprofloxacin), and PLA–Dox (doxycycline). Inhibition zone diameters are expressed in millimeters.

The distributions of inhibition zone diameters for individual clinical isolates are shown in Figure 6.

**Figure 7:**
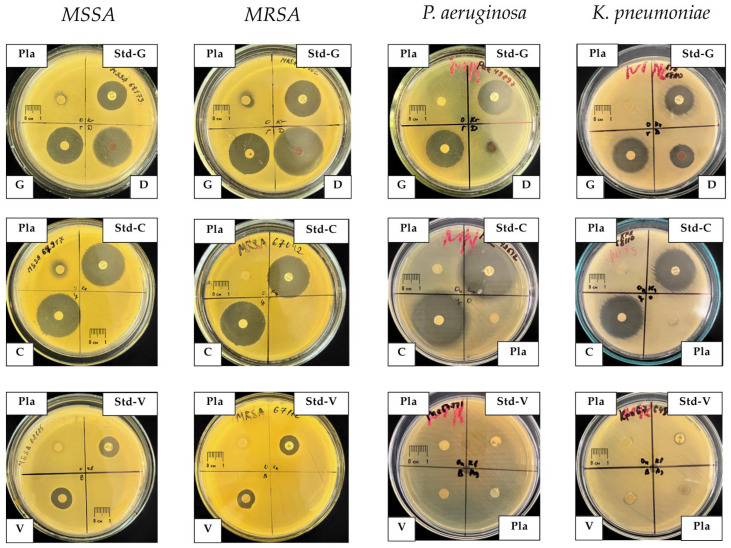
Representative inhibition zones around PLA-based composite disks and commercial antibiotic disks against clinical isolates of methicillin-susceptible *Staphylococcus aureus* (MSSA), methicillin-resistant *Staphylococcus aureus* (MRSA), *Klebsiella pneumoniae*, and *Pseudomonas aeruginosa* after 24 h of incubation at 37 °C. Disks are labeled as follows: Pla—neat PLA (negative control); G—PLA–gentamicin; C—PLA–ciprofloxacin; D—PLA–doxycycline; V—PLA–vancomycin; Std-G, Std-C, and Std-V—commercial gentamicin, ciprofloxacin, and vancomycin disks (positive controls), respectively. Clear halos around the disks indicate zones of bacterial growth inhibition.

Comparison of the activity of PLA composites with standard commercial antibiotic disks demonstrated preservation of the antimicrobial activity of antibiotics after incorporation into the polymer matrix and thermal processing. Representative examples of growth inhibition zones for clinical isolates are shown in Figure 7.

## 3. Discussion

Despite substantial advances in systemic antibiotic therapy, implant-associated infections remain among the most difficult-to-manage complications in orthopedic and trauma practice [17]. The formation of bacterial biofilms on implant surfaces markedly reduces the effectiveness of systemically administered antibiotics and promotes persistence and recurrence of infection [5,8,18,19]. In this context, the development of implantable materials with local antimicrobial activity is considered a promising approach for creating a temporary “antibacterial barrier” at the implantation site during the critical early postoperative period [20,21].

In the present study, PLA-based composites modified with antibiotics from different pharmacological classes demonstrated pronounced antimicrobial activity against clinically relevant pathogens. These findings support the fundamental feasibility of using biodegradable polymer matrices as platforms for local antibiotic therapy in orthopedic and trauma settings associated with a high risk of infectious complications [22]. Importantly, the obtained results extend beyond the demonstration of efficacy of an individual material and provide a basis for a more universal, platform-oriented approach.

A key conceptual aspect of this work is the comparative evaluation of antibiotics from different pharmacological classes—an aminoglycoside (gentamicin), a glycopeptide (vancomycin), a tetracycline (doxycycline), and a fluoroquinolone (ciprofloxacin)—within a unified PLA platform. Such a design allows the biodegradable polymer matrix to be considered not merely as a passive carrier, but as an engineerable framework for tailoring differentiated profiles of local antibacterial protection of implants according to the anticipated microbiological risk [23].

The use of clinical isolates alongside ATCC reference strains substantially enhances the clinical relevance of the obtained data. MRSA and MSSA were selected as leading causative agents of postoperative infections, characterized by high adhesive capacity and a pronounced propensity for biofilm formation [2], which is supported by the high prevalence of biofilm-forming phenotypes and adhesin-associated genes among clinical *S. aureus* isolates [24]. In contrast, *Pseudomonas aeruginosa* and *Klebsiella pneumoniae* reflect the contribution of Gram-negative nosocomial flora with a high prevalence of multidrug resistance, as evidenced by numerous reviews on the current global dissemination of MDR strains and the mechanisms underlying their resistance [25,26]. The assembled panel of microorganisms reproduces the spectrum of pathogens determining the clinical outcomes of implant-associated infections, allowing the present results to be regarded as more representative of real clinical conditions compared with studies limited exclusively to laboratory strains [27].

Comparative analysis of the antimicrobial activity of PLA composites against ATCC reference strains and clinical isolates revealed substantially higher interstrain variability of responses in clinical microbial populations. While ATCC strains are characterized by high reproducibility of antibiotic susceptibility and are widely used as standardized model organisms for primary screening of antimicrobial materials, clinical isolates reflect the actual microbiological diversity of the hospital environment. They are characterized by heterogeneous resistance mechanisms, variable expression of efflux systems, and differences in their ability to adhere to and form biofilms on the surfaces of medical materials, which fundamentally distinguishes them from laboratory strains used in simplified in vitro models [28,29,30]. The comparison of reference strains with clinical isolates not only serves to validate the experimental model, but also enables identification of the limitations associated with translating laboratory findings into clinical practice [28].

It should be noted that small apparent inhibition zones were occasionally observed around neat PLA disks when tested against certain clinical staphylococcal isolates. Since PLA does not possess intrinsic antibacterial activity, this effect is most likely associated with minor local environmental changes at the polymer–agar interface inherent to the agar diffusion assay, such as slight pH shifts caused by initial hydrolysis of PLA or local alterations in moisture conditions. Such effects may influence Gram-positive bacteria to a greater extent than Gram-negative organisms such as *Pseudomonas aeruginosa*, which possess more robust outer membrane barriers and stress-response mechanisms. Importantly, this phenomenon was observed only in a subset of clinical isolates and was not detected for the standardized ATCC strains, which further supports its association with interstrain variability rather than intrinsic antimicrobial properties of PLA.

The observed differences between ATCC reference strains and clinical isolates, manifested as shifts in bacterial susceptibility levels and increased interstrain variability of antimicrobial responses, indicate the limited translatability of data obtained exclusively using laboratory reference cultures to real-world clinical scenarios of implant-associated infections. The use of ATCC strains alone may lead to a systematic overestimation of the antimicrobial potential of polymer-based local delivery systems, as these strains typically do not reflect the complex resistance phenotypes characteristic of pathogen populations circulating in hospital settings [28,29]. This underscores the necessity of incorporating clinical isolates into preclinical evaluation protocols for antibacterial implantable materials.

In view of the identified interstrain variability, it becomes critically important to consider the dependence of composite efficacy on the pharmacological profile of the incorporated antibiotic. The present results demonstrate that the antimicrobial activity of PLA composites is determined not so much by the mere fact of antibiotic incorporation into the polymer matrix, but rather by the antimicrobial spectrum and mechanism of action of the specific agent, as well as by the microbiological characteristics of the target pathogen, including its susceptibility phenotype and the presence of resistance mechanisms to the respective antibiotics [30]. This finding precludes universalization of the concept of a “one-size-fits-all antibacterial PLA implant” and emphasizes the need for rational antibiotic selection based on the anticipated microbiological profile of infection and the antimicrobial susceptibility patterns of strains circulating in a given healthcare setting. In a broader context, biodegradable polymer matrices should be regarded as engineerable platforms for local antimicrobial therapy, whose functional properties are shaped by the interplay between polymer characteristics and the pharmacological properties of the incorporated antibiotic [31].

An important factor underlying these differences in antimicrobial performance is the release behavior of antibiotics from the PLA matrix, which determines the local concentration of active agents at the implantation site. The release profiles demonstrated pronounced differences depending on molecular weight, hydrophilicity, and the nature of interactions between antibiotic molecules and the PLA matrix.

Gentamicin exhibited rapid and nearly complete release (>90% within 48 h), which can be attributed to its high hydrophilicity and weak interactions with the hydrophobic PLA matrix. Ciprofloxacin demonstrated a more sustained release profile without reaching a plateau within 4 days, likely due to partial intermolecular interactions with the polymer matrix that slow diffusion.

Vancomycin showed limited release, which can be explained by its high molecular weight (~1485 Da) and steric constraints on diffusion through the polymer network. Doxycycline exhibited the lowest release, possibly due to strong intermolecular interactions (e.g., hydrogen bonding) with PLA, as well as its known sensitivity to thermal processing. Although direct evidence of degradation was not obtained, partial structural modification cannot be excluded.

An apparently contradictory observation was noted for doxycycline: despite showing the lowest cumulative release in the spectrophotometric assay, the PLA–doxycycline composite still exhibited relatively pronounced antibacterial activity in the agar diffusion test. This discrepancy can be explained by the different principles of the two methods. The release study reflects the cumulative amount of drug transferred into the bulk liquid medium, whereas the agar diffusion assay depends primarily on the local concentration of biologically active molecules released from the sample surface into the surrounding agar.

Because doxycycline is active at relatively low concentrations, even a limited released fraction may be sufficient to generate a measurable inhibition zone. Moreover, inhibition zone formation is governed by local concentration gradients and diffusion behavior in the agar, rather than the total amount of antibiotic released into the bulk medium.

Therefore, the absence of a direct correlation between cumulative release and inhibition zone diameter is not unexpected and reflects the fundamental methodological differences between bulk release measurements and diffusion-based biological assays. These results indicate that low cumulative release does not necessarily preclude a significant local antibacterial effect.

The observed release behavior is consistent with FTIR data indicating physical incorporation of antibiotics and possible intermolecular interactions, as well as with TGA results showing that processing temperatures were below the onset of major thermal degradation.

Overall, antibiotic release from PLA-based systems is governed by a combination of diffusion, molecular size, and intermolecular interactions, which should be considered when designing controlled-release materials.

In this context, the observed release behavior is linked to the antibacterial performance of the composites in diffusion-based assays, as inhibition zone formation depends on both antibiotic activity and its ability to diffuse from the polymer matrix. Accordingly, agar diffusion results should be interpreted with consideration of both release kinetics and diffusion behavior.

In addition to diffusion behavior, antibiotic loading is another important parameter influencing antimicrobial performance. Higher loading levels may increase the amount of drug released and enlarge inhibition zones, whereas lower loadings may result in insufficient local concentrations. In this study, a standardized loading of 5 wt.% was used to ensure comparability between different antibiotics.

Although the actual loading (encapsulation) efficiency was not directly quantified in this study, the successful incorporation of antibiotics into the PLA matrix is supported by a combination of physicochemical and functional data, as discussed above. In particular, FTIR and TGA analyses, together with UV–Vis release studies, indicate the presence and functional availability of the incorporated antibiotics. Taken together, these findings provide consistent indirect evidence of effective antibiotic incorporation into the PLA matrix. Quantitative determination of loading efficiency requires additional analytical studies.

The most pronounced and reproducible antimicrobial activity was observed for PLA–ciprofloxacin composites, which were effective against both Gram-positive and Gram-negative clinical pathogens, including *Pseudomonas aeruginosa* and *Klebsiella pneumoniae*. The high activity of fluoroquinolones against Gram-negative bacteria, mediated by inhibition of DNA gyrase and topoisomerase IV [32], in combination with efficient diffusion of ciprofloxacin from the polymer matrix [33], likely underlies the formation of large inhibition zones in the applied experimental model. In the clinical context, this indicates a high potential of PLA–ciprofloxacin composites in scenarios involving a risk of implant contamination by nosocomial Gram-negative flora.

PLA–gentamicin composites also exhibited a stable broad spectrum of activity, including against clinical isolates of MRSA, *P. aeruginosa*, and *K. pneumoniae*. These findings are consistent with accumulated clinical experience with aminoglycosides incorporated into bone cements and local antibiotic depots [34,35] and support the translational relevance of extending this clinically validated concept to biodegradable polymer carriers, in which PLA may be considered a functional alternative to non-resorbable materials while maintaining comparable antimicrobial efficacy.

In contrast to broad-spectrum antibiotics, PLA–vancomycin composites exhibited selective activity predominantly against *Staphylococcus aureus*, including methicillin-resistant clinical isolates, which is consistent with the well-known pharmacological limitations of glycopeptides [36,37]. Vancomycin remains a drug of choice for the treatment of MRSA infections and is recommended in international clinical guidelines as a cornerstone agent for the management of severe staphylococcal infections. This highlights the importance of aligning the antibiotic spectrum with the anticipated microbiological profile of infection and points to the potential of PLA–vancomycin composites as a specialized local prophylactic strategy against staphylococcal infections.

PLA–doxycycline composites demonstrated pronounced activity against clinical *Staphylococcus aureus* isolates but substantially reduced efficacy against *Pseudomonas aeruginosa*, which is consistent with the known limited susceptibility of this pathogen to tetracyclines due to the barrier function of the outer membrane and active efflux systems [38,39]. This finding further underscores the necessity of context-dependent antibiotic selection when designing antibacterial polymer-based systems.

From a practical perspective, an important outcome of this study is the demonstration that antimicrobial activity of antibiotics is preserved after incorporation into the PLA matrix and subsequent thermal processing at temperatures relevant to extrusion and additive manufacturing. This is of critical importance for the development of 3D-printable antibacterial biodegradable materials, as elevated temperatures are traditionally regarded as a major risk factor for degradation of biologically active molecules. The antimicrobial activity of PLA composites, evaluated alongside standard antibiotic susceptibility testing disks, indicates preservation of functional antibiotic activity following processing. It should be noted that commercial antibiotic disks are designed for rapid antibiotic release under standardized conditions, whereas the PLA-based composites represent a sustained-release system. Therefore, the disks serve primarily as qualitative reference controls rather than directly comparable systems.

As highlighted in recent reviews, most studies in the field of 3D-printable biodegradable implants have focused primarily on enhancing osteoconductive and mechanical properties, whereas the deliberate introduction of pronounced antimicrobial functionality remains fragmented and is often limited to proof-of-concept demonstrations [15,40]. In this context, the present comparative approach, combining a clinically relevant panel of strains with analysis of antibiotics from different pharmacological classes within a unified platform, addresses a substantial gap in the existing literature.

The obtained results allow PLA composites loaded with antibiotics to be considered as elements of a tunable antibacterial platform that can potentially be adapted to the microbiological profile of a specific healthcare institution or patient cohort. Given the marked variability in hospital flora composition and antimicrobial resistance levels between institutions, the possibility of targeted antibiotic selection for modification of implantable materials acquires particular practical relevance and opens the prospect of transitioning from a “one implant fits all” paradigm toward the development of context-dependent antibacterial implants [41].

Within this framework, an important perspective for further development is the incorporation of antibiotic combinations with complementary mechanisms of action within a single PLA matrix. The gentamicin–vancomycin combination is particularly attractive due to its potential synergistic activity against *Staphylococcus aureus*, including MRSA, as well as its broader antimicrobial coverage, with vancomycin targeting Gram-positive pathogens and gentamicin extending activity toward Gram-negative bacteria. Similar combinations have already been successfully employed in antibiotic-loaded bone cements for the prevention of implant-associated infections [42]. However, translation of this concept to biodegradable PLA matrices will require careful optimization of antibiotic loading and release kinetics, as interactions between antibiotics may influence elution profiles. This represents an important direction for future research on multifunctional PLA-based antibacterial implants.

It should be considered that local antibiotic delivery at sub-inhibitory concentrations in peripheral zones of the implant may create selective pressure conducive to the emergence of resistant microbial populations, as previously reported for various local antibacterial implant systems and coatings [43]. This emphasizes the need to optimize release profiles and dosing regimens of active components when designing such platforms.

Another important aspect relevant to the clinical translation of PLA-based antibiotic composites is the selection of an appropriate terminal sterilization method. In the present study, composite disks were sterilized by UV irradiation prior to in vitro antimicrobial testing in order to minimize potential alterations of the polymer matrix and the incorporated antibiotics. However, for future preclinical (in vivo) studies and potential clinical applications, clinically established sterilization procedures should be considered.

Among the available approaches, gamma irradiation, ethylene oxide (EtO) sterilization, and electron-beam (e-beam) irradiation are widely used for biodegradable polymeric medical devices, including PLA-based systems. Gamma irradiation (typically in the range of 10–30 kGy) represents one of the most commonly applied terminal sterilization methods for implantable biomaterials. EtO sterilization is particularly suitable for thermosensitive materials due to its low operating temperature and high penetration capacity, whereas electron-beam irradiation provides a rapid alternative to gamma sterilization, often associated with shorter exposure times. Nevertheless, radiation-based sterilization may induce polymer chain scission, reduction in molecular weight, and alterations in degradation behavior, which can subsequently influence drug release kinetics and mechanical properties of the material. Therefore, the influence of sterilization on both polymer stability and antibiotic activity should be systematically evaluated in future studies on PLA-based antibacterial implants [44,45].

It should also be noted that the present study was conducted under in vitro conditions and does not account for the influence of proteins, cellular components, and dynamic conditions of biological fluids, which may substantially modify antibiotic release profiles and biological activity [43]. Although in vitro release of antibiotics from the PLA matrix was evaluated in the present study, the obtained data represent simplified conditions and do not fully reflect the complexity of the in vivo environment. Accordingly, future studies should focus on detailed quantitative characterization of release kinetics under physiologically relevant conditions, evaluation of the ability of PLA–antibiotic composites to prevent biofilm formation and interfere with early stages of biofilm development on material surfaces, modeling of biofilm systems, and in vivo validation of the antimicrobial efficacy of the developed composites.

## 4. Materials and Methods

### 4.1. Materials

Polylactic acid (PLA, NatureWorks, Plymouth, MN, USA) was used as the polymer matrix. Gentamicin sulfate, vancomycin hydrochloride, doxycycline hyclate, and ciprofloxacin hydrochloride (Sigma-Aldrich, St. Louis, MO, USA) were employed as antibacterial agents. All solvents and reagents were of analytical grade and were used without further purification. Mueller–Hinton agar (MHA, HiMedia, Mumbai, India) was used for microbiological assays.

### 4.2. Preparation of PLA/Antibiotic Composites

PLA-based composite materials containing different antibiotics were prepared according to a unified processing protocol to ensure valid comparative analysis of their antibacterial activity. In all formulations, PLA constituted 95 wt.% of the composite, while the respective antibiotic (gentamicin sulfate, vancomycin hydrochloride, doxycycline hyclate, or ciprofloxacin) accounted for 5 wt.%. Antibiotics were incorporated into the PLA matrix at a fixed concentration of 5 wt.%, selected based on literature precedents for antibiotic-loaded orthopedic biomaterials, including PMMA bone cements and biodegradable polymer systems, as well as preliminary formulation experiments indicating that this loading provides measurable antimicrobial activity while preserving the processability of the PLA matrix during thermal processing [46,47].

To facilitate homogeneous mixing, PLA pellets were first ground into a fine powder using a laboratory knife mill (Retsch GRINDOMIX GM 200, Haan, Germany). The obtained PLA powder and the corresponding antibiotic powders were then premixed using a mortar and pestle for 5 min at room temperature to promote preliminary dispersion of the antibiotic within the polymer matrix.

The obtained mixtures were subjected to hot pressing using a laboratory thermal press (Blue Press X Pneu, Schulze GmbH, Berlin, Germany) at 160–180 °C, corresponding to the processing window of PLA and approximating extrusion and additive manufacturing conditions. The material was pressed a for 30 s. To ensure complete densification while minimizing prolonged thermal exposure of the antibiotics, the pressing cycle was repeated up to three times, allowing the material to cool briefly between cycles. This procedure resulted in the formation of dense and visually homogeneous composite plates.

After pressing, the plates were allowed to cool to room temperature on a metal surface. Cylindrical disks with a diameter of 6 mm and a thickness of approximately 300 μm were then punched out from the plates using a sterile hollow punch. These dimensions correspond to the standard sample format used in agar diffusion assays.

Prior to microbiological testing, the composite disks were sterilized by ultraviolet (UV) irradiation for 30 min to eliminate potential surface contamination.

### 4.3. Physicochemical Characterization

#### 4.3.1. Morphology (SEM)

The surface morphology of the PLA-based composite filaments was examined by scanning electron microscopy (SEM) using a Helios 5 CX dual-beam SEM/FIB system (Thermo Fisher Scientific, Waltham, MA, USA).

SEM imaging was performed under high-vacuum conditions using an accelerating voltage of 3 kV and a beam current of 100 pA. Micrographs were acquired using a secondary electron (SE) detector (Thermo Fisher Scientific, Waltham, MA, USA) at different magnifications to evaluate both the overall surface morphology and the distribution of microstructural features within the polymer matrix. For each sample, multiple regions were examined at different magnifications to ensure representativeness of the observed morphology.

#### 4.3.2. Chemical Structure (FTIR)

Fourier transform infrared (FTIR) spectroscopy was performed using a Nicolet iS10 spectrometer (Thermo Fisher Scientific, Waltham, MA, USA). Spectra were recorded in the range of 4000–400 cm^−1^. The analysis was conducted to identify characteristic functional groups and assess possible interactions between the PLA matrix and incorporated antibiotics.

#### 4.3.3. Thermal Analysis (TGA)

Thermogravimetric analysis (TGA) was carried out using a STA 6000 thermal analyzer (PerkinElmer, Shelton, CT, USA). Measurements were performed under an inert atmosphere in the temperature range from 30 to 600 °C at a heating rate of 10 °C/min. Samples with a mass of approximately 10–20 mg were placed in open alumina crucibles.

Thermogravimetric (TG) and derivative thermogravimetric (DTG) curves were recorded to evaluate the thermal stability and degradation behavior of the samples. The onset degradation temperature (Tonset) was determined as the temperature corresponding to the initial deviation from the baseline. The temperature of the maximum degradation rate (Tmax) was determined from the DTG peak. The residual mass was recorded at 600 °C.

#### 4.3.4. In Vitro Release Study

The release behavior of antibiotics from PLA-based composites was investigated under in vitro conditions using polymer discs fabricated by thermal pressing. All samples had a uniform geometry with a diameter of 6.0 mm and a thickness of 0.30 mm. The antibiotic loading was fixed at 5 wt.% for all compositions.

The average mass of the discs depended on the incorporated antibiotic and was as follows: gentamicin—6.26 ± 0.32 mg, ciprofloxacin—9.67 ± 0.57 mg, vancomycin—7.32 ± 0.29 mg, and doxycycline—7.49 ± 0.43 mg.

Release experiments were performed under conditions of partial medium replacement. Ten discs of each composition were immersed in phosphate buffer solution at a ratio of 10 μL per 1 mg of sample mass. The experiments were carried out in triplicate for each antibiotic.

Aliquots of 0.1 mL were withdrawn at 24 h intervals over a period of 4 days. After each sampling, an equal volume of fresh buffer was added to maintain sink conditions.

The concentration of released antibiotics was determined by UV–Vis spectrophotometry, with sample preparation procedures adapted to the physicochemical properties of each compound. Representative UV–Vis spectra of antibiotic solutions at different concentrations are provided in the Supplementary Materials (Figure S2).

For gentamicin, which lacks a distinct chromophore in the UV–Vis region, a derivatization method was employed. A 100 μL aliquot of the sample was mixed with 0.5 mL of ascorbic acid solution in dimethyl sulfoxide (DMSO) (25 mg/mL), and the volume was adjusted to 5 mL with DMSO. The mixture was heated in a water bath for 45 min, resulting in the formation of a colored product. Absorbance was measured at 365 and 510 nm.

For vancomycin, ciprofloxacin, and doxycycline, which possess intrinsic chromophores, analysis was performed without derivatization. Sample aliquots (0.1 mL) were diluted with phosphate buffer (20–100-fold, depending on the expected concentration) to ensure measurements within the linear range of the calibration curves. Absorbance was recorded at the following wavelengths: 297 nm (vancomycin), 323 nm (ciprofloxacin), and 352 nm (doxycycline).

Calibration curves were constructed for each antibiotic by plotting absorbance versus concentration.

The cumulative amount of released antibiotic was calculated by accounting for both the drug removed during previous samplings and the amount remaining in the release medium at each time point. The total released amount at time $t_{n}$ ($m_{total,n}$) was calculated using the following equation:

$$m_{total,n}=\sum_{i=1}^{n}C_{i}\cdot V_{sample}+C_{n}\cdot V_{medium}$$

where $C_{i}$ is the concentration of the antibiotic in the sampled aliquot at time $t_{i}$, $V_{sample}$ is the volume of the withdrawn sample, and $V_{medium}$ is the total volume of the release medium.

### 4.4. Microorganisms and Culture Conditions

The antibacterial activity of the composites was evaluated against reference strains *Staphylococcus aureus* ATCC 29313, *Pseudomonas aeruginosa* ATCC 27853, and *Escherichia coli* ATCC 25922. For comparative assessment of the antimicrobial efficacy of the PLA composites, clinical isolates provided by the research laboratory of Karaganda Medical University (Kazakhstan) were used. The study included methicillin-resistant *S. aureus* (MRSA) (*n* = 10), methicillin-susceptible *S. aureus* (MSSA) (*n* = 10), *Pseudomonas aeruginosa* (*n* = 10), and *Klebsiella pneumoniae* (*n* = 10).

All samples were anonymized, and no patient personal data were used in the study. Prior to the experiments, the isolates were stored at −80 °C. Before testing, the strains were retrieved from storage and subcultured on nutrient media, specifically blood agar supplemented with 5% sheep blood. Incubation was performed for 24 h at 37 °C.

### 4.5. Preparation of Microbial Inoculum

Microbial suspensions were prepared in sterile physiological saline and standardized to 0.5 McFarland units (1.5 × 10^8^ CFU/mL) in accordance with commonly accepted recommendations for the disk diffusion method of antimicrobial susceptibility testing (EUCAST) [48], using a densitometer (DEN-1, Biosan, Riga, Latvia).

### 4.6. Antimicrobial Activity Assay (Agar Diffusion Method)

The antibacterial activity of the composites was determined using the agar diffusion (disk diffusion) method in accordance with EUCAST guidelines [48]. Petri dishes containing Mueller–Hinton agar were uniformly inoculated with the prepared microbial suspension using a sterile swab. PLA composite disks were placed on the agar surface and gently pressed to ensure close contact. Incubation was carried out at 37 °C for 24 h [48].

Negative and positive controls were included in each experiment. Disks made of neat PLA without antibiotics were used as negative controls. Standard commercial antibiotic susceptibility testing disks (BIOANALYSE Limited, Turkey) were used as positive controls: gentamicin (10 µg/disk), ciprofloxacin (5 µg/disk), and vancomycin (5 µg/disk). Comparison of inhibition zones formed by composite disks and standard antibiotic disks provided a qualitative assessment of antibacterial activity, and a comparison of inhibition zones formed by composite disks and standard antibiotic disks provided a qualitative assessment of antibacterial activity.

After incubation, the diameters of growth inhibition zones were measured in millimeters using a calibrated vernier caliper (Stayer, Pinto, Madrid, Spain; resolution 0.01 mm). For ATCC reference strains, each experiment was performed in three independent replicates. For evaluation of antimicrobial activity against clinical isolates, 10 independent clinical strains of each bacterial species were used, allowing interstrain variability in susceptibility to be taken into account. For descriptive interpretation of the experimental data, a conditional activity scale was applied: <10 mm—no activity; 10–15 mm—low activity; 15–20 mm—moderate activity; >20 mm—pronounced activity. Similar descriptive classifications of inhibition zone diameters have been reported in disk diffusion studies, including investigations of plant-derived antimicrobials and bioactive systems, where zone diameters are categorized to facilitate comparative interpretation of antimicrobial effects [49,50,51].

### 4.7. Statistical Analysis

Statistical analysis was performed using GraphPad Prism 10 (GraphPad Software, San Diego, CA, USA). All experiments with ATCC reference strains were performed in triplicate (*n* = 3), while antibacterial activity against clinical isolates was evaluated using ten independent isolates per group (*n* = 10 for MSSA, MRSA, *Klebsiella pneumoniae*, and *Pseudomonas aeruginosa*).

Normality of data distribution was assessed using the Shapiro–Wilk test. As most datasets deviated from normal distribution, non-parametric statistical methods were applied. Comparisons among multiple composite formulations were performed using the Kruskal–Wallis test followed by Dunn’s post hoc test with Holm correction for multiple comparisons.

Data are presented as mean ± standard deviation (mean ± SD). Differences were considered statistically significant at *p* < 0.05.

## 5. Conclusions

In the present study, a comparative evaluation of the antimicrobial efficacy of PLA-based composites modified with antibiotics from different pharmacological classes (gentamicin, ciprofloxacin, doxycycline, and vancomycin) was performed for the first time within a unified PLA platform against ATCC reference strains and clinical isolates associated with implant-associated and healthcare-associated infections. It was demonstrated that incorporation of antibiotics into the polylactic acid matrix followed by thermoplastic processing does not result in a loss of their biological activity, thereby confirming the technological feasibility of this approach for the development of antibacterial biodegradable materials potentially suitable for extrusion and 3D printing of implantable constructs.

Comparative analysis revealed a clear dependence of the antimicrobial activity of PLA composites on the pharmacological class of the antibiotic and the susceptibility spectrum of the target microorganisms. Ciprofloxacin- and gentamicin-based composites exhibited the broadest and most reproducible activity profiles against clinically relevant Gram-positive and Gram-negative pathogens, whereas vancomycin and doxycycline displayed the expected selectivity of action. These findings support the context-dependent nature of antibacterial functionalization of PLA and underscore the necessity of pharmacologically informed antibiotic selection based on the anticipated microbiological risk.

The inclusion of clinical isolates alongside ATCC reference strains enabled demonstration of higher interstrain variability in susceptibility and highlighted the limited translatability of data obtained exclusively from laboratory models to real clinical settings. Despite the inherent limitations of the in vitro model the obtained physicochemical characterization and in vitro release data support the structural integrity of the composites and their ability to provide controlled antibiotic release, while the results overall confirm the promise of PLA as an adaptable platform for local antibiotic delivery for the prevention of implant-associated infections and provide a foundation for further preclinical investigations.

## Figures and Tables

**Table 1:** Mean diameters of inhibition zones (mm) ± SD against ATCC reference strains (*n* = 3 independent replicates).

Sample	*S. aureus* ATCC 29313	*P. aeruginosa* ATCC 27853	*E. coli* ATCC 25922
PLA	7.0 ± 1.0	6.0 ± 0.0	6.5 ± 1.08
PLA–Gen	25.0 ± 1.0	28.7 ± 0.58	24.7 ± 0.58
PLA–Cip	31.3 ± 0.58	34.7 ± 0.58	43.0 ± 1.73
PLA–Dox	26.0 ± 1.53	7.0 ± 1.0	26.3 ± 0.58
PLA–Van	16.0 ± 1.0	6.7 ± 1.73	6.3 ± 0.58
Gentamicin	20.0 ± 1.0	23.0 ± 1.0	20.3 ± 0.58
Ciprofloxacin	31.0 ± 1.0	33.0 ± 0.0	42.7 ± 1.53
Vancomycin	16.3 ± 0.58	6.0 ± 0.0	7.7 ± 0.58

**Table 2:** Antibacterial activity of PLA-based composites and standard antibiotic disks against clinical isolates (mean inhibition zone diameter ± SD, mm; n = 10).

Sample	MSSA	MRSA	*K. pneumoniae*	*P. aeruginosa*
PLA	11.1 ± 3.31	9.8 ± 3.55	7.0 ± 1.18	6.3 ± 0.32
PLA–Gen	25.5 ± 1.18	25.7 ± 2.71	23.9 ± 2.18	26.8 ± 1.48
PLA–Cip	29.7 ± 1.77	30.9 ± 3.73	32.0 ± 2.45	35.9 ± 2.28
PLA–Dox	26.3 ± 2.71	27.4 ± 3.98	17.8 ± 4.44	10.2 ± 1.48
PLA–Van	14.7 ± 0.67	15.4 ± 0.84	7.4 ± 1.26	6.2 ± 0.63
Gentamicin	21.3 ± 1.34	21.9 ± 2.73	19.8 ± 1.23	22.2 ± 1.40
Ciprofloxacin	28.5 ± 2.32	29.3 ± 3.47	29.6 ± 2.59	34.7 ± 1.34
Vancomycin	15.15 ± 1.06	15.2 ± 1.14	7.2 ± 0.92	6.0 ± 0.0

## Data Availability

The data presented in this study are included in the article. Further inquiries can be directed to the corresponding authors.

## Supplementary Materials

The following supporting information can be downloaded at: https://www.mdpi.com/article/10.3390/antibiotics15040373/s1, Figure S1: TGA/DTA curves of the antibiotics used in this study: ciprofloxacin (Cip), vancomycin (Van), doxycycline (Dox), and gentamicin (Gen). The thermograms illustrate the thermal stability and decomposition behavior of each compound, providing a basis for assessing their suitability for incorporation into PLA under thermoplastic processing conditions; Table S1: Thermal parameters obtained from TGA analysis of PLA and PLA–antibiotic composites; Figure S2: UV–Vis spectra of antibiotic solutions at different concentrations: (A) gentamicin after reaction with ascorbic acid, (B) ciprofloxacin, (C) vancomycin, and (D) doxycycline. The spectra were used for calibration and quantitative analysis of antibiotic concentration in release studies.
