# Supplementary material for: Antibiotic-Loaded PLA Composites for Local Prevention of Implant-Associated Infections: Comparative Evaluation Against Reference Strains and Clinical Isolates
Source: Antibiotics (Basel). 2026 Apr 6;15(4):373. doi: 10.3390/antibiotics15040373 (PMC13113662; doi:10.3390/antibiotics15040373)
Supplement: Supplementary file 1 [file antibiotics-15-00373-s001.zip › antibiotics-4196221-supplementary.pdf]

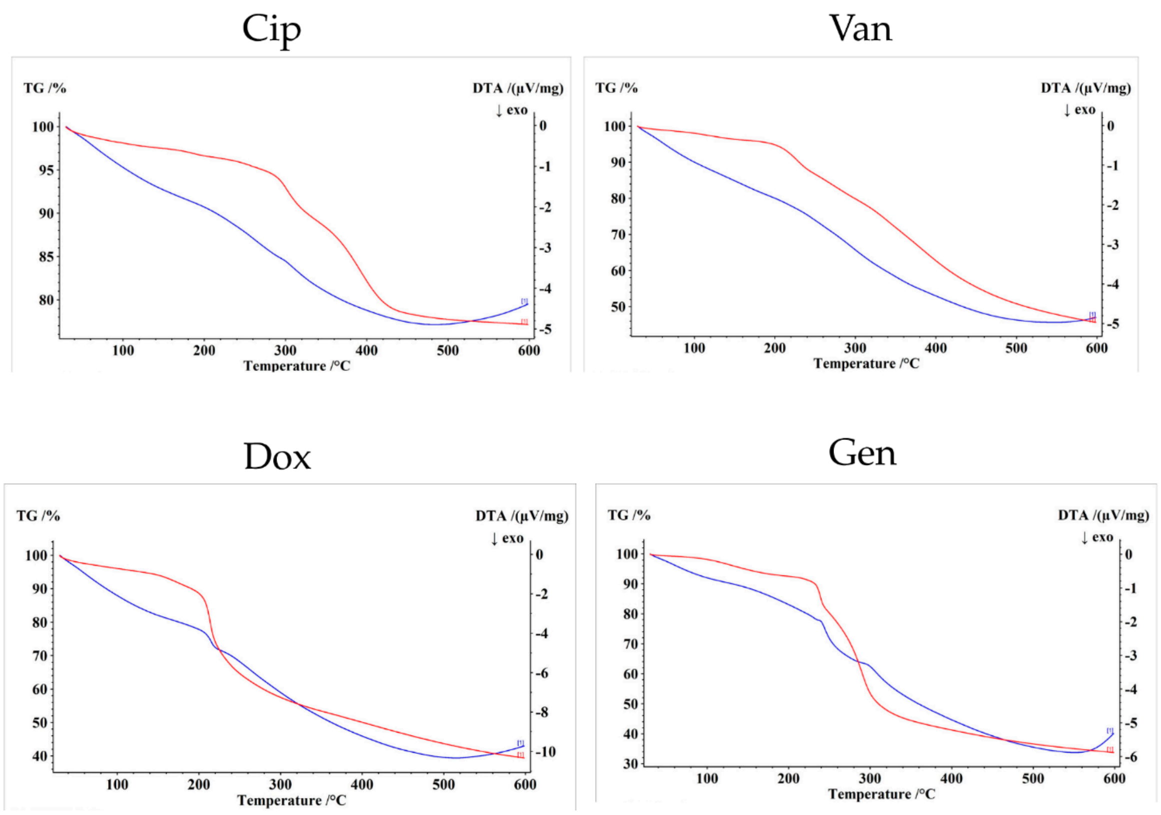

Figure S1. TGA/DTA curves of the antibiotics used in this study: ciprofloxacin (Cip), vancomycin (Van), doxycycline (Dox), and gentamicin (Gen). The thermograms illustrate the thermal stability and decomposition behavior of each compound, providing a basis for assessing their suitability for incorporation into PLA under thermoplastic processing conditions.

Table S1. Thermal parameters obtained from TGA analysis of PLA and PLA–antibiotic composites.

Sample	Tonset (°C)	T5% (°C)	Tmax (°C)	Residue (%)
**PLA**	300	331	348	0.1
**PLA–CIP**	290	307	345	3.6
**PLA–DOX**	288	306	343	1.9
**PLA–GEN**	285	305	344	1.3
**PLA–VAN**	278	296	340	0.0

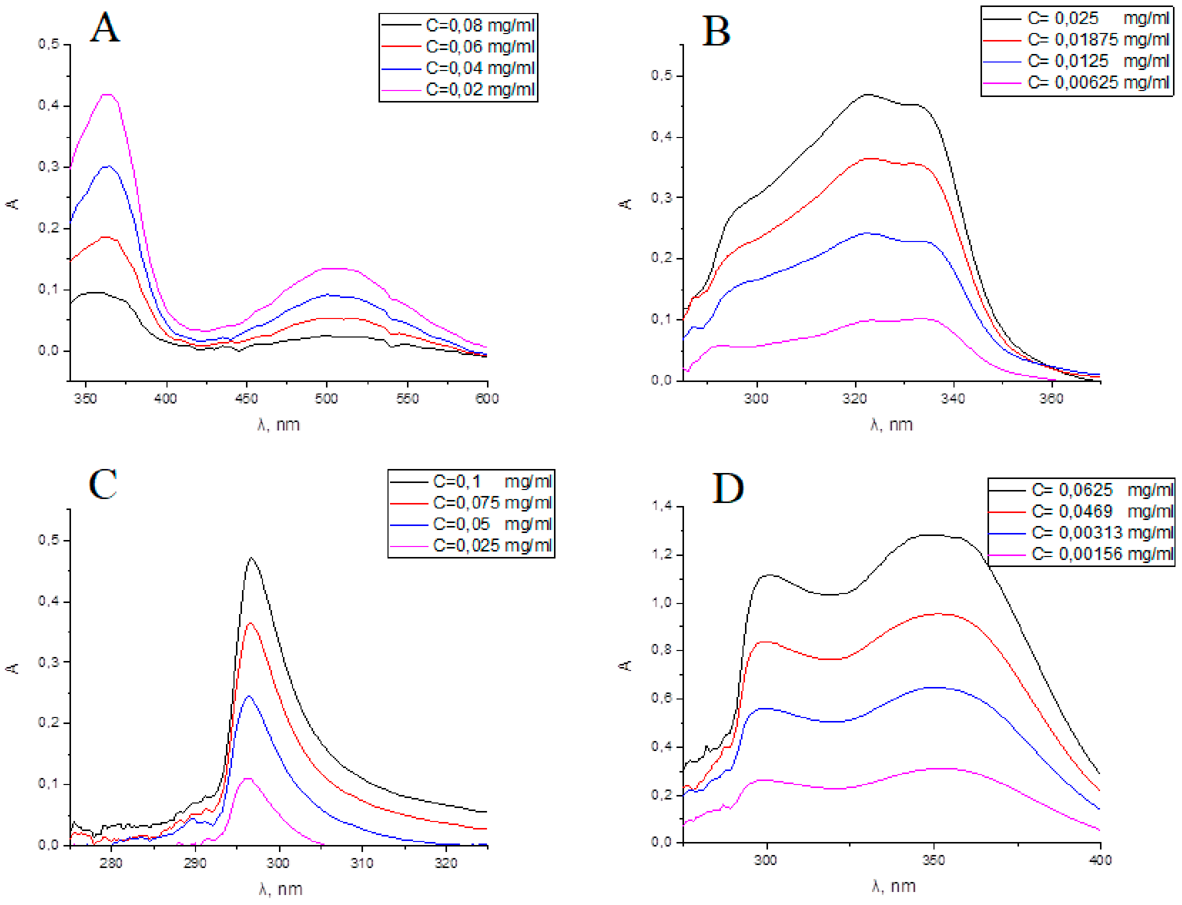

Figure S2. UV–Vis spectra of antibiotic solutions at different concentrations: (A) gentamicin after reaction with ascorbic acid, (B) ciprofloxacin, (C) vancomycin, and (D) doxycycline. The spectra were used for calibration and quantitative analysis of antibiotic concentration in release studies.
